# Manipulation of Host Cholesterol by Obligate Intracellular Bacteria

**DOI:** 10.3389/fcimb.2017.00165

**Published:** 2017-05-05

**Authors:** Dhritiman Samanta, Minal Mulye, Tatiana M. Clemente, Anna V. Justis, Stacey D. Gilk

**Affiliations:** Department of Microbiology and Immunology, Indiana University School of MedicineIndianapolis, IN, USA

**Keywords:** cholesterol, lipid raft, *Chlamydia*, *Coxiella*, *Rickettsia*, *Anaplasma*, lipid droplet

## Abstract

Cholesterol is a multifunctional lipid that plays important metabolic and structural roles in the eukaryotic cell. Despite having diverse lifestyles, the obligate intracellular bacterial pathogens *Chlamydia, Coxiella, Anaplasma, Ehrlichia, and Rickettsia* all target cholesterol during host cell colonization as a potential source of membrane, as well as a means to manipulate host cell signaling and trafficking. To promote host cell entry, these pathogens utilize cholesterol-rich microdomains known as lipid rafts, which serve as organizational and functional platforms for host signaling pathways involved in phagocytosis. Once a pathogen gains entrance to the intracellular space, it can manipulate host cholesterol trafficking pathways to access nutrient-rich vesicles or acquire membrane components for the bacteria or bacteria-containing vacuole. To acquire cholesterol, these pathogens specifically target host cholesterol metabolism, uptake, efflux, and storage. In this review, we examine the strategies obligate intracellular bacterial pathogens employ to manipulate cholesterol during host cell colonization. Understanding how obligate intracellular pathogens target and use host cholesterol provides critical insight into the host-pathogen relationship.

## Introduction

To establish the intracellular niche, obligate intracellular pathogens must overcome numerous obstacles. First, the pathogen must recognize and bind to their target host cell. Second, they must penetrate the host cell plasma membrane, most often by tricking the host cell into engulfing the pathogen through phagocytosis. Once inside the target cell, the pathogen initially resides in a membrane-bound compartment called the phagosome that, under normal conditions, progressively acidifies to a mature phagolysosome. While some bacteria can thrive in this environment, others prevent fusion between the phagosome and lysosome, or escape the phagosome and replicate in the host cytosol. For vacuolar pathogens, the pathogen-containing vacuole must protect from the host innate immune the bacteria system, while at the same time allowing access to nutrients required for bacterial growth. In order to establish these highly specialized intracellular niche, pathogens manipulate host gene expression, metabolism, or trafficking pathways.

In contrast to facultative intracellular or extracellular pathogens, obligate intracellular pathogens rely on the host cell for a large percentage of their growth requirements. As a result, obligate intracellular pathogens have sophisticated mechanism to manipulate the host cell to obtain essential nutrients. One of the targeted host cell factors is cholesterol, a major lipid component of eukaryotic membranes that strongly influences membrane structure and function. Structurally, cholesterol affects membrane fluidity and permeability, with higher cholesterol levels increasing membrane rigidity. Cholesterol concentrates in membrane microdomains known as lipid rafts, which are specialized signaling platforms involved in signal transduction (Simons and Toomre, 2000). Further, intracellular cholesterol is a critical player in Golgi trafficking (Stüven et al., 2003), endocytic trafficking (van der Kant et al., 2013), and intra-organelle membrane contact sites (Eden et al., 2016). Pathogenic bacteria target cholesterol not only to gain entry to host cells but also to hijack host cell signaling pathways favorable for intracellular survival. This review discusses the role of cholesterol in host-pathogen interactions, from the perspective of obligate intracellular bacterial pathogens that reside in membrane-bound compartments (*Chlamydia, Coxiella, Anaplasma, and Ehrlichia*) or in the host cell cytoplasm (*Rickettsia*).

## Intracellular lifestyles

### *Chlamydia* spp.

*Chlamydia* are obligate intracellular bacteria that cause trachoma (*C. trachomatis* serovars Ab, B, Ba, C), urogenital tract infections (*C. trachomatis* serovars D-K), lymphogranuloma venereum (*C. trachomatis* serovar L1, L2, L3) and pneumonia (*C. pneumoniae* and *C. psittaci*). *Chlamydia* have a biphasic life cycle, alternating between the infectious elementary body (EB) and the replicative reticulate body (RB). Following EB attachment to the host cell, the *Chlamydia* Type III secretion system (T3SS) injects proteins into the host cell cytosol, inducing actin rearrangement and bacterial uptake (Clifton et al., 2004). Once internalized, the bacteria block phagosome maturation and reside in a membrane-bound compartment known as the inclusion. The inclusion membrane contains both host and bacterial proteins and is non-fusogenic with endosomes and lysosomes, but intercepts nutrient-rich Golgi-derived vesicles and multivesicular bodies in the recycling pathway (Hackstadt et al., 1995, 1996; Heinzen et al., 1996; Beatty, 2006). Within the inclusion, EBs differentiate into RBs, which replicate and eventually differentiate back to EBs prior to host cell egress and reinfection. During this infectious cycle, *Chlamydia* T3SS effector proteins are translocated across the inclusion membrane and into the host cytosol, where they manipulate host pathways to divert nutrients such as amino acids, lipids, and iron to the inclusion (reviewed in Bastidas et al., 2013).

### Coxiella burnetii

*Coxiella burnetii* is the causative agent of human Q fever, an aerosol-borne zoonotic disease characterized by flu-like symptoms during acute infection and endocarditis in chronically infected patients. Following phagocytosis of the environmentally stable *C. burnetii* small cell variant (SCV), the *C. burnetii*-containing phagosome matures through the endocytic pathway to a phagolysosome (Howe and Mallavia, 2000; Howe et al., 2003). The acidic pH of the phagolysosome activates *C. burnetii* metabolism and differentiation to the replicative form known as the large cell variant (LCV) (Hackstadt and Williams, 1981). At this point, fusion between the *C. burnetii* parasitophorous vacuole (PV) and host endosomes, lysosomes, and autophagosomes creates a large, phagolysosome-like vacuole that promotes bacterial replication (reviewed in Voth and Heinzen, 2007). *C. burnetii* actively manipulates host cell functions such as apoptosis and vesicular trafficking by secreting effector proteins into the cytoplasm through the Dot/Icm Type IV secretion system (T4SS) (reviewed in Moffatt et al., 2015).

### *Anaplasma phagocytophilum* and *Ehrlichia chaffeensis*

*Anaplasma phagocytophilum* and *Ehrlichia chaffeensis* are tick-borne obligate intracellular bacterial pathogens belonging to the family Anaplasmataceae. *A. phagocytophilum*, the causative agent of human granulocytic anaplasmosis, infects human neutrophils and multiplies within vacuoles called inclusions or morulae. The *A. phagocytophilum* inclusion does not contain endosomal or lysosomal markers, although it interacts with the nutrient-rich endocytic and autophagic pathways (Webster et al., 1998; Mott et al., 1999; Niu et al., 2006). Like *C. burnetii*, the *A. phagocytophilum* T4SS effector proteins modulate host cell processes including autophagosome formation and SUMOylation (Niu et al., 2010; Al-Khedery et al., 2012; Beyer et al., 2015).

*Ehrlichia chaffeensis* exclusively infects human monocytes and macrophages and causes human monocytic ehrlichiosis. Similar to *A. phagocytophilum* inclusions, *E. chaffeensis* inclusions do not contain late endosomal markers and only weakly stain for lysosomal vacuolar ATPase (Barnewall et al., 1997). However, in contrast to *A. phagocytophilum, E. chaffeensis* inclusions are labeled with early endosomal markers, suggesting the *E. chaffeensis* resides in a vacuolar niche similar to an early endosome (Mott et al., 1999). In addition, the *E. chaffeensis* T4SS effector protein Etf-1 induces autophagy and redirects nutrients to the inclusion to support *E. chaffeensis* intracellular growth (Lin et al., 2016).

### *Rickettsia* spp.

*Rickettsia* spp. are small coccobacilli and the causative agents of Rocky Mountain Spotted Fever (*R. rickettsia*), Mediterranean spotted fever (*R. conorii*), louse-borne typhus (*R. prowazekii*), and scrub typhus (*Orientia tsutsugamushi*). Most commonly transmitted to humans by arthropods, *Rickettsia* spp. infect and replicate in the endothelial cells of blood vessels and major organs. Following uptake by the host cell, rickettsial phospholipase degrades the phagosomal membrane, releasing the bacteria into the cytoplasm where they replicate freely (Walker et al., 2001). Several species of *Rickettsia* spread from cell to cell by reorganizing and mobilizing host cell actin (Heinzen et al., 1993), while *O. tsutsugamushi* escapes the host cell through a process similar to viral budding (Ogawa et al., 2014).

## Role of cholesterol in bacterial entry and exit

Cholesterol-rich microdomains in the plasma membrane, known as lipid rafts, play a key role during pathogen attachment and entry into host cells. Lipid rafts are enriched in cholesterol, with 3- to 5-fold more cholesterol than the surrounding membrane (Pike, 2003). Lipid rafts also have higher concentrations of sphingolipids and signal transduction proteins such as integrins, kinases, phosphatases, and G protein-coupled receptors (Brown and Rose, 1992; Sargiacomo et al., 1993; Chun et al., 1994; Lisanti et al., 1994; Gorodinsky and Harris, 1995; Liu et al., 1996; Mineo et al., 1996; Bickel et al., 1997). Caveolae, a morphologically distinct subset of lipid rafts with a flask-shaped structure, contain cholesterol-binding caveolin proteins involved in clathrin-independent endocytosis (Chang et al., 1992; Stang et al., 1997; Orlandi and Fishman, 1998; Schubert et al., 2001; Lai, 2003). As lipid rafts are key components of cell signaling and endocytosis, bacterial pathogens often target lipid rafts during host cell entry. Hijacking raft-associated signaling proteins promotes internalization of intracellular bacteria and subsequent development of the intracellular niche.

### *Chlamydia* spp.

The role of lipid rafts during *Chlamydia* host cell entry has been controversial. Early studies found that *C. trachomatis* serovar L2 and *C. psittaci* strain GPIC was associated with lipid rafts during infection of HeLa cells, and cholesterol depletion using methyl-beta-cyclodextrin (MβCD) inhibited bacterial entry in a dose-dependent manner (Jutras et al., 2003). Disrupting lipid rafts with the cholesterol-binding compounds filipin and nystatin impaired entry of *C. pneumoniae, C. psittaci*, and *C. trachomatis* serovars E, F, and K (but not A, B, and C) into HeLa cells (Stuart et al., 2003) and serovar K into J774 macrophages (Norkin et al., 2001). Further, although caveolin was not necessary for entry, these strains co-localized with the major caveolae protein caveolin-1 (Norkin et al., 2001; Stuart et al., 2003). Recent work suggests *C. pneumoniae* uptake by HL cells, but not attachment, also involves lipid rafts (Korhonen et al., 2012). Similarly, Gabel reported that MβCD cholesterol extraction inhibited entry of *C. trachomatis* serovars D, E, K, and L2 into HeLa cells (Gabel et al., 2004). However, disrupting lipid raft function with filipin, nystatin, or antibodies against cholera toxin B bound to lipid raft GM1 ganglioside had no effect on infection (Gabel et al., 2004). This study also did not find bacteria associated with lipid rafts or caveolin. Further, GPI-anchored proteins, which are enriched in lipid rafts, were not required for *C. trachomatis* entry into CHO cells (Jutras et al., 2003). In support of these findings, *C. trachomatis* serovar L2 entry into cholesterol-free DHCR24^−/−^ mouse embryonic fibroblasts was identical to cholesterol supplemented cells (Gilk et al., 2013).

The discrepancies between these studies may be attributed to differences in bacterial isolates, host cell type, or pleotropic effects on membrane function by MβCD (Zidovetzki and Levitan, 2007). It is also possible that multiple, redundant entry pathways exist using different host cell receptors. Recent studies have shown that two *C. pneumoniae* proteins, adhesin CPn0473 and invasin Pmp21, bind to host epidermal growth factor receptor (EGFR) and trigger lipid raft-mediated uptake (Mölleken et al., 2013; Fechtner et al., 2016). *C. trachomatis* also adhered to the lipid raft protein EphrinA2 receptor (EphA2) (Chakraborty et al., 2012; Subbarayal et al., 2015), and the *C. trachomatis* invasin protein Ctad1 bound to β1 integrin in lipid rafts during internalization (Stallmann and Hegemann, 2016). Targeting multiple host cell lipid raft receptors may broaden host cell tropism and increase the potential for successful host cell colonization.

### C. burnetii

During host cell entry, *C. burnetii* utilizes α_V_β_3_ integrin (Capo et al., 1999), a transmembrane protein found in lipid rafts (Triantafilou and Triantafilou, 2003). In cholesterol-free DHCR24^−/−^ fibroblasts, *C. burnetii* internalization decreased almost 90% compared to DHCR24^−/−^ fibroblasts supplemented with exogenous cholesterol (Gilk et al., 2013). Blocking α_V_β_3_ with antibodies or the α_V_β_3_ ligand vitronectin further reduced internalization in DHCR24^−/−^ fibroblasts, reinforcing the significance of lipid rafts in *C. burnetii* internalization. Targeting the α_V_β_3_ pathway may be a strategy employed by *C. burnetii* to evade the host immune response. α_V_β_3_ normally functions during macrophage phagocytosis of apoptotic cells (Wu et al., 2005), a process that suppresses the immune response by inhibiting macrophage production of interleukin (IL)-1β, IL-8, tumor necrosis factor (TNF)- α, leukotriene C_4_, and thromboxane B2 (Fadok et al., 1998). Thus, the α_V_β_3_-dependent pathway may enable *C. burnetii* to enter host cells without eliciting an inflammatory response.

### *A. phagocytophilum* and *E. chaffeensis*

*A. phagocytophilum* and *E. chaffeensis* have also been reported to utilize lipid rafts or caveolae for host cell entry (Lin and Rikihisa, 2003b). Similar to *Chlamydia* spp., cholesterol depletion using MβCD, or lipid raft disruption with nystatin or NBD-cholesterol, blocked infection by both *A. phagocytophilum* and *E. chaffeensis* (Lin and Rikihisa, 2003b). Removing host surface GPI-anchored proteins with phosphoinositide phospholipase C (PI-PLC) also inhibited entry of both bacterial species, suggesting lipid raft-associated GPI-anchored proteins are necessary for infection. Depletion of plasma membrane caveolae with cholera toxin B reduced *A. phagocytophilum* and *E. chaffeensis* entry by 90%, and both GM1 and caveolin-1 localized to early inclusions (Lin and Rikihisa, 2003b). These data suggest that *A. phagocytophilum* and *E. chaffeensis* utilize caveolae-mediated endocytosis for host cell entry.

### *Rickettsia* spp.

Lipid rafts are involved in both host cell entry and exit of different *Rickettsia* spp. MβCD extraction of plasma membrane cholesterol blocked *R. conorii* uptake (Martinez et al., 2005). Further, the *R. conorii* outer membrane protein OmpB bound the host protein Ku70, a subunit of DNA-dependent protein kinase (DNA-PK) which is found in the nucleus and plasma membrane lipid rafts (Koike, 2002; Lucero et al., 2003; Martinez et al., 2005). Ku70 co-localized with *R. conorii* attached to Vero or HeLa cells, and an antibody directed against the extracellular N-terminus of Ku70 decreased *R. conorii* entry (Martinez et al., 2005). Thus, *R. conorii* OmpB facilitates host cell entry by binding to the lipid raft-associated host protein Ku70. In addition to OmpB, *R. conorii* OmpA bound the lipid raft integrins α_2_ and β_1_ on the cell surface (Upla et al., 2004; Hillman et al., 2013). While the signaling cascade triggered by Ku70 is not known, it may mimic integrin signaling and lead to actin-mediated phagocytosis (Martinez et al., 2005).

*O. tsutsugamushi* was found in lipid rafts of infected cells, suggesting the bacteria associate with lipid rafts (Kim et al., 2013). However, the inability of MβCD or filipin to impair *O. tsutsugamushi* host cell entry indicates that cholesterol or lipid rafts are not involved (Kim et al., 2013). While caveolin did not associate directly with the bacteria, the *O. tsutsugamushi* protein HtrA co-localized with caveolin during host cell exit, a process where the bacteria causes the plasma membrane to bulge out similar to viral budding (Kim et al., 2013). Thus, it appears that plasma membrane cholesterol is not involved in *O. tsutsugamushi* entry but plays a key role during bacterial egress from host cells.

Targeting lipid rafts is clearly an important strategy by obligate intracellular pathogens, with *Chlamydia* spp., *C. burnetii, A. phagocytophilum, E. chaffeensis*, and *R. conorii* all utilizing lipid rafts during host cell entry. Lipid rafts serve as signaling platforms to trigger host cell actin rearrangement and phagocytosis, thus allowing the bacteria to subvert host cell machinery to gain entry. Further, caveolin-mediated entry may facilitate development of the intracellular niche for vacuolar pathogens. For example, *Chlamydia* inclusion membrane caveolin may facilitate direct interception of Golgi-derived exocytic vesicles that are rich in sphingolipids and other nutrients essential for *Chlamydia* growth (Hackstadt et al., 1996; Van Ooij et al., 2000). While the presence of caveolin on the *C. burnetii* PV has not been explored, caveolin most likely plays an important role during host cell colonization by *Chlamydia* spp., *A. phagocytophilum*, and *E. chaffeensis*.

## Cholesterol-rich microdomains on pathogen-containing vacuoles

While the functions are less understood, cholesterol-rich microdomains are also found on the membrane of bacteria-containing vacuoles.

### *Chlamydia* spp.

Filipin labeling of *C. trachomatis*-infected HeLa cells revealed cholesterol-rich microdomains on the inclusion membrane (Carabeo et al., 2003; Mital et al., 2010). These microdomains co-localized with four chlamydial inclusion membrane proteins (IncB, Inc101, Inc222, and Inc850) and active host Src-family kinases (SFKs) (Mital et al., 2010). SFKs were not required for Inc microdomain formation, leaving the relationship between SFKs and Inc proteins unclear (Mital and Hackstadt, 2011). It is not known whether the Incs are recruited to these inclusion microdomains, or if the Inc proteins themselves trigger microdomain formation. Inc222 and Inc850 stably interact with one another and may form a complex within the inclusion microdomains. While the functions of IncB, Inc101, and Inc222 are unknown, Inc850 may play a critical role during inclusion development by mediating microtubule-dependent trafficking of the nascent chlamydial inclusion to the perinuclear microtubule organizing center (MTOC) (Campbell et al., 1989a,b; Grieshaber et al., 2003). Inc850 was found to bind dynein light chain, DYNLT1, thus facilitating interactions between the inclusion and the host microtubule network that are required for trafficking to the MTOC (Mital et al., 2010, 2015). Inclusion trafficking to the MTOC is thought to promote homotypic fusion between inclusions, which serves as a potential mechanism to share nutrients or exchange genetic material (Richards et al., 2013). *Chlamydia* clinical strains that cannot undergo homotypic fusion cause less severe disease with fewer recoverable bacteria, suggesting this is an important virulence factor (Geisler et al., 2001). With the recent development of genetic tools in *Chlamydia*, the role of Inc850 in inclusion trafficking and virulence can now be tested.

In addition to the Incs, the host SFKs Fyn and Src localized to cholesterol-rich microdomains of *C. trachomatis* and *C. pneumoniae* inclusions but not the rodent species *C. caviae* and *C. muridarum* (Mital et al., 2010; Mital and Hackstadt, 2011). The SFKs are non-receptor membrane-associated tyrosine kinases that regulate microtubule-dependent trafficking by phosphorylating tubulin and binding to dynein-associated proteins (Macurek et al., 2008; Colello et al., 2010; Levi and Shalgi, 2010). SFK activity was necessary for dynein-dependent trafficking of the nascent *C. trachomatis* inclusion to the MTOC (Mital et al., 2010). In addition, Fyn was involved in sphingomyelin acquisition by *Chlamydia*, most likely through microtubule-dependent trafficking (Mital and Hackstadt, 2011). *Chlamydia* species that do not recruit SFKs to their inclusion membranes do not traffic to the MTOC but have increased inclusion development and bacterial growth in SFK-deficient cells, suggesting SFKs restrict growth of some *Chlamydia* species (Mital et al., 2010; Mital and Hackstadt, 2011).

### C. burnetii

The *C. burnetii* PV membrane is sterol-rich and contains the lipid raft proteins flotillin-1 and flotillin-2 (Howe and Heinzen, 2006). It is not known, however, if there are organized microdomains on the PV membrane, or if these proteins play a role during *C. burnetii* infection. Like *C. trachomatis*, multiple *C. burnetii* PVs in a host cell will fuse, though there is no evidence for microtubule-dependent trafficking of the PV and the role of homotypic fusion in pathogenesis is not known.

Cholesterol-rich microdomains have been found on the pathogen-containing vacuoles of both *C. trachomatis* and *C. burnetii*, though the function has only been explored in *C. trachomatis*. Cholesterol or other sterols are also enriched in the *A. phagocytophilum* inclusion (Xiong et al., 2009). In addition to mediating microtubule-dependent trafficking as in the case of *C. trachomatis* inclusion, cholesterol may play a structural role in the membranes of pathogen-containing vacuoles. For example, high cholesterol membranes are more rigid and may create a stronger physical barrier between the pathogen and host cell defenses. Further, cholesterol regulates proteins involved in endosomal trafficking and fusion, and could facilitate recruitment of nutrient-rich endosomes to support bacterial growth.

## Cholesterol trafficking to pathogen-containing vacuoles

Endogenous cholesterol is synthesized in the endoplasmic reticulum (ER) and trafficked to the plasma membrane before distribution throughout the cell. The major source of exogenous cholesterol is through receptor-mediated uptake of cholesterol bound to low density lipoprotein (LDL). LDL particles are internalized by clathrin-mediated endocytosis and transported through the endocytic pathway to lysosomes, where cholesterol esters are hydrolyzed to free cholesterol for cellular use. Regardless of the source, cholesterol is transported throughout the cell by both vesicular and non-vesicular (e.g., cholesterol transport proteins and membrane contact sites) trafficking pathways.

### *Chlamydia* spp.

While the bacteria lack the machinery to synthesize cholesterol, it is found in the *C. trachomatis* membrane in addition to inclusion microdomains (Wylie et al., 1997; Hatch and McClarty, 1998; Stephens et al., 1998; Carabeo et al., 2003). *Chlamydia* appears to actively acquire host cholesterol, as inhibiting bacterial protein synthesis with chloramphenicol drastically decreased inclusion cholesterol levels (Carabeo et al., 2003). This suggests that bacterial proteins, possibly secreted into the host cytoplasm, directly manipulate host cholesterol trafficking pathways. Both *de novo* synthesized and exogenous LDL-derived cholesterol trafficked to *C. trachomatis* inclusion through a microtubule-dependent process and involved transit through the Golgi apparatus before delivery to the inclusion membrane (Carabeo et al., 2003). However, Golgi-dependent cholesterol trafficking was not essential for *Chlamydia* replication, and cholesterol-rich multivesicular bodies (MVBs) also deliver cholesterol to the inclusion (Carabeo et al., 2003; Beatty, 2006, 2008). The MVB pathway may be essential, as disruption of the MVB trafficking decreased cholesterol in the *C. trachomatis* inclusion, delayed inclusion maturation, and reduced bacterial growth (Beatty, 2006, 2008). Interestingly, *C. trachomatis* inclusion formation and bacterial growth was unaffected in cholesterol-free DHCR24^−/−^ mouse embryonic fibroblasts, suggesting cholesterol precursors may be sufficient for *C. trachomati*s infection (Gilk et al., 2013).

In addition to Golgi-dependent and MVB trafficking, a third mechanism of cholesterol transport to the inclusion utilizes the high-density lipoprotein (HDL) biogenesis machinery involved in cholesterol efflux. HDL is formed when cholesterol and phospholipids are transported to extracellular ApoA-1 by the lipid binding proteins ATP-binding cassette transporters A1 and G1 (ABCA1, ABCG1), and CLA1. Intriguingly, while ABCA1, CLA1, and ApoA-1 localized to the inclusion membrane, both CLA1 and ApoA-1 were also found in discrete foci within the inclusion lumen (Cox et al., 2012). siRNA knockdown of ABCA1 or pharmaceutical inhibitors of ABCA1 and CLA1 transporter activity significantly reduced *C. trachomatis* growth (Cox et al., 2012). While the mechanism is not clear, it is possible that *C. trachomatis* diverts these proteins to the inclusion as a mechanism to obtain cholesterol.

### C. burnetii

The *C. burnetii* PV is sterol-rich, although filipin labeling does not indicate that cholesterol or other sterols are present in the *C. burnetii* envelope (Howe and Heinzen, 2006). Despite encoding two unique eukaryote-like sterol reductase homologs (Seshadri et al., 2003; Beare et al., 2009; Gilk et al., 2013), *C. burnetii* does not appear to synthesize cholesterol and instead obtains cholesterol from the host cell. Cholesterol-rich MVBs fuse with the PV (Gilk et al., 2013) and both endogenous and LDL-derived cholesterol traffic to the PV through unknown pathways (Mulye et al., 2017). *C. burnetii* also interacts with host cholesterol trafficking by recruiting the host cholesterol-binding protein ORP1L (oxysterol binding protein related protein 1 long) to the PV in a T4SS-dependent manner (Justis et al., 2017). ORP1L is an endosome/lysosome-localized Rab7 effector protein that serves two conformation-dependent functions in host cells (Rocha et al., 2009). When bound to cholesterol, ORP1L takes on a compact conformation, allowing Rab7-RILP to interact with dynein motors and direct minus-end transport of endosomes along microtubules. Alternately, when not bound to cholesterol, ORP1L is in an extended conformation and binds to the VAP proteins on the ER, participating in endosome/lysosome-ER membrane contact sites (Rocha et al., 2009; van der Kant et al., 2013). Interestingly, while the *C. burnetii* PV membrane is sterol-rich, fluorescent co-localization and electron microscopy suggest that ORP1L participates in PV-ER contact sites and is likely not binding cholesterol on the PV membrane (Justis et al., 2017). ORP1L was required for optimal *C. burnetii* PV expansion possibly through ORP1L-dependent trafficking to the PV (Justis et al., 2017). ORP1L may also be involved in transfer of cholesterol from the PV to the ER, similar to the proposed role of ORP1L during adenovirus infection (Cianciola et al., 2013).

Inhibiting host cell cholesterol metabolism disrupted PV morphology and bacterial replication, demonstrating that cholesterol and other sterols play an important role in *C. burnetii* infection (Howe and Heinzen, 2006). Although cholesterol itself was not essential for *C. burnetii* infection of cholesterol-free DHCR24^−/−^ fibroblasts (Gilk et al., 2013), increasing PV cholesterol through cholesterol supplementation or pharmacological inhibitors was bactericidal (Mulye et al., 2017). Intriguingly, *C. burnetii* death was due to increased PV acidification when PV cholesterol levels were elevated (Mulye et al., 2017). The proton pump vATPase plays a key role in lysosomal acidification and has been localized to the *C. burnetii* PV (Heinzen et al., 1996). vATPase activity is affected by lysosomal membrane cholesterol (Cox et al., 2007), leading to the possibility that cholesterol levels regulate PV pH through vATPase.

### A. phagocytophilum

Cholesterol is an important component of the *A. phagocytophilum* and *E. chaffeensis* cell envelopes, providing physical integrity in the absence of typical bacterial lipid A and peptidoglycan (Lin and Rikihisa, 2003b). Cholesterol is also enriched on the *A. phagocytophilum* inclusion membrane and lumen (Xiong et al., 2009). Like *Chlamydia* and *C. burnetii, A. phagocytophilum* lacks genes for cholesterol biosynthesis and recruits host cholesterol by targeting cholesterol trafficking (Lin and Rikihisa, 2003a). *A. phagocytophilum*-infected cells have increased LDL uptake, resulting in 2-fold more cholesterol than uninfected cells (Xiong et al., 2009). LDL-derived cholesterol was trafficked to the *A. phagocytophilum* inclusion by the host cell protein NPC1 (Niemann-Pick disease, type C1), a cholesterol-binding membrane protein required for cholesterol transfer from endosomes and lysosomes to the ER (Karten et al., 2009). Within 24 h of infection, *A. phagocytophilum* recruited NPC1 to the inclusion membrane via NPC1-positive, LAMP1/2-negative vesicles (Xiong and Rikihisa, 2012). Bacterial protein synthesis was required for NPC1 recruitment, and bacterial growth was significantly impaired in NPC1-deficient cells (Xiong and Rikihisa, 2012). Furthermore, vesicle-associated membrane protein 4 (VAMP4) and syntaxin 16, which participate in LDL-cholesterol transport from NPC1 vesicles to the Golgi network, were also recruited to the *A. phagocytophilum* inclusion (Xiong and Rikihisa, 2012). Thus, *A. phagocytophilum* hijacks NPC1-mediated LDL trafficking as a method to divert cholesterol to the bacterial inclusion for incorporation into the bacterial envelope and inclusion membrane.

Given that cholesterol plays important roles in both the bacteria and vacuole membranes, it is no surprise that obligate intracellular pathogens have devised multiple ways to recruit host cell cholesterol. Cholesterol binding proteins, which serve to move cholesterol between membranes, localize to the vacuoles of both *C. trachomatis* and *C. burnetii*. Cholesterol-rich MVBs also fuse with the *C. trachomatis* and *C. burnetii* vacuoles, and LDL trafficking of internalized extracellular cholesterol is important for *C. trachomatis, C. burnetii*, and *A. phagocytophilum*. In the case of *A. phagocytophilum*, the bacteria target a specific subset of LDL-positive vesicles, while the underlying mechanism of LDL trafficking to the *C. trachomatis* inclusion is not known. Finally, the promiscuous fusogenicity of the *C. burnetii* PV may account for LDL delivery to this vacuole. Most likely, other undiscovered pathways also deliver cholesterol to the bacteria, all serving to provide this important resource to the intracellular niche.

## Bacterial modification of cholesterol

While not possessing the full biosynthetic machinery to generate cholesterol *de novo*, both *C. trachomatis* and *C. burnetii* express cholesterol-modifying enzymes which may modify host cell cholesterol or cholesterol precursors.

### C. trachomatis

The *C. trachomatis* gene CT149 is a putative carboxylic esterase containing a cholesterol recognition consensus sequence and two GXSXG cholesterol esterase motifs (Peters et al., 2012). Further, recombinant CT149 exhibited cholesterol esterase activity *in vitro*, and CT149 ectopic expression in HeLa cells decreased cholesterol ester levels, with a corresponding increase in free cholesterol (Peters et al., 2012). Antibodies generated against CT149 localized this protein to the bacteria and not the host cytoplasm, suggesting CT149 functions inside the inclusion (Peters et al., 2012). Interestingly, lipid droplets (LDs), which store cholesterol esters, were observed inside the chlamydial inclusion (Cocchiaro et al., 2008), presenting the possibility that CT149 liberates cholesterol from LDs for use by the bacteria.

### C. burnetii

*C. burnetii* expresses two eukaryotic-like sterol reductase enzymes, CBU1158 and CBU1206 (Seshadri et al., 2003; Beare et al., 2009). CBU1206 has homology to Δ24 sterol reductases, which function in the final step of mammalian cholesterol or yeast ergosterol synthesis (Beare et al., 2009). Heterologous expression of CBU1206 in *Saccharomyces cerevisae* functionally complements deletion of the yeast Δ24 sterol reductase *erg4*, indicating CBU1206 can modify sterols (Gilk et al., 2010). However, if CBU1206 modifies cholesterol in *C. burnetii* or host cells is unknown. Further, CBU1206 contains nine predicted transmembrane domains and is most likely in the *C. burnetii* cell envelope, which raises interesting questions about when and where CBU1206 might modify host sterols (Gilk et al., 2010). The enzymatic capability for the putative *C. burnetii* Δ7 sterol reductase CBU1158 remains unexplored. Determining the importance of the *C. burnetii* putative sterol reductases during infection, along with their substrate specificity and location of action, will be critical to understanding the role of cholesterol and other sterols during *C. burnetii* infection.

## Manipulating cholesterol homeostasis

Eukaryotic cells tightly regulate cholesterol levels by balancing metabolism (biosynthesis and breakdown), uptake, efflux, and storage. *De novo* biosynthesis occurs in the ER, with the conversion of HMG-CoA to mevalonate by HMG-CoA reductase (HMGR) being the rate-limiting step. Cellular cholesterol levels can also be increased through uptake of cholesterol bound to LDL via the LDL receptor (LDLR). Both biosynthesis and uptake are regulated at the expression level by sterol regulatory element-binding protein (SREBP) or liver X receptor (LXR) transcription factors, which increase transcription of HMGR and LDLR under low cholesterol conditions. When cellular cholesterol levels are high, cholesterol can be transported out of the cell (i.e., effluxed), broken down into bile acids or steroids, or esterified and stored in LDs. *Chlamydia* spp., *C. burnetii*, and *A. phagocytophilum* all target host cholesterol homeostasis, particularly at the level of gene transcription.

### Cholesterol metabolism and uptake

#### *Chlamydia* spp.

*C. pneumoniae* has been linked to atherosclerosis, a disease that results from dyslipidemia, or increased levels of circulating lipids. In mice, peripheral blood monocytes can spread *C. pneumoniae* from the lung to the liver, a key site of lipid metabolism (Moazed et al., 1998; Marangoni et al., 2006). A recent *in vivo* study found that *C. pneumoniae*-infected mice had decreased hepatic bile acid levels and increased serum cholesterol levels, as compared to uninfected or *C. trachomatis*-infected mice (Marangoni et al., 2015). These changes have been linked to Cyp7a1 (cholesterol 7α-hydroxylase), a host enzyme that catalyzes cholesterol breakdown into bile acids. Along with two transcription factors involved in regulating Cyp7a1 expression, *lxr-*α and *srebp1c, cyp7a1*was downregulated in *C. pneumoniae-*infected liver cells (Marangoni et al., 2015). A separate study reported a dose-dependent decrease in *cyp7a1* promoter activity in *C. pneumoniae*-infected human hepatocytes (Michelini et al., 2012). Together, these data suggest that through Cyp7a1, *C. pneumoniae* downregulates cholesterol catabolism in liver cells. *C. pneumoniae* also altered uptake of serum cholesterol in the mouse liver, by downregulating *ldlr* expression and upregulating expression of *idol* (inducible degrader of the LDLR) (Marangoni et al., 2015). By decreasing cholesterol catabolism in the liver, while also decreasing cholesterol uptake, *C. pneumoniae* infection may lead to increased circulating cholesterol and promote atherosclerosis.

In contrast to liver cells, *C. pneumoniae* increased LDL uptake in infected human monocyte-derived macrophages (Kalayoglu and Byrne, 1998). This led to formation of foam cells, which are lipid-laden cells important in atherosclerosis progression. LDL uptake also increased approximately 2.5-fold in *C. pneumoniae*-infected human monocytes and human umbilical vein epithelial (HUVEC) cells (Yoshida et al., 2006; Evani and Ramasubramanian, 2016). In HUVEC cells, this was partially due to increased expression of cholesterol uptake receptors, including scavenger receptor A, LOX-1, and CD36, which all internalize oxidized low density lipoprotein (oxLDL) (Yoshida et al., 2006; Campbell et al., 2013; Sun et al., 2014). Interestingly, glycan from *C. pneumoniae*, but not *C. trachomatis*, bound and activated LOX-1 (Campbell et al., 2013). Recombinant *C. pneumoniae* Hsp60 also increased LOX-1 expression in endothelial cells of hypercholesterolemic rabbits, further suggesting that the bacteria actively interact with the LOX-1 pathway to increase LDL uptake (Lin et al., 2011).

Less is known about *C. trachomatis* manipulation of cholesterol metabolism. In human trophoblasts, *C. trachomatis* downregulated *hmgr*, leading to lower levels of cholesterol and the cholesterol-derived steroids estrogen and progesterone; this is hypothesized to impair trophoblast implantation and placentation during pregnancy (Azenabor et al., 2007). Although not the primary target organ, *C. trachomatis* has been found in mouse liver lesions and can cause perihepatitis in humans (Barteneva et al., 1996). Gene expression profiles in *C. trachomatis-*infected mouse livers reported upregulation of *lxr*α*, lxr*β, and *cyp7a1*, suggesting cholesterol catabolism is elevated (Marangoni et al., 2015). *C. trachomatis* downregulated *ldlr* in human HepG2 hepatocellular cells, suggesting the bacteria decreases cholesterol uptake (Bashmakov et al., 2010). Cholesterol biosynthesis appears to be critical for *C. trachomatis* infection, as the HMGR inhibitor mevastatin reduced *C. trachomatis* growth (Bashmakov et al., 2010). The requirement for *de novo* host synthesized cholesterol would suggest sterol intermediates or cholesterol metabolites are important for *C. trachomatis* infection of liver cells.

#### C. burnetii

*C. burnetii*-infected Vero cells had a 73% increase in cellular cholesterol at 6 days post-infection, as compared to mock infected cells (Howe and Heinzen, 2006). Pharmaceutical inhibitors of either HMGR or LDL uptake blocked PV formation and bacterial growth, and both *hmgr* and *ldlr* were upregulated during *C. burnetii* infection of Vero cells (Howe and Heinzen, 2006). Intriguingly, this upregulation was not observed until 4 days post-infection, when the *C. burnetii* PV is large and the bacteria are in logarithmic growth. However, at 6 days post-infection the expression levels returned to the same level as mock-infected cells (Howe and Heinzen, 2006) indicating a temporal regulation of cholesterol biosynthesis and uptake. Supporting this hypothesis, *C. burnetii* PV size and growth were found most sensitive to cholesterol levels only during the first 2 days of infection (Mulye et al., 2017). As elevated cholesterol in the PV is bacteriolytic, it is possible that *C. burnetii* reduces PV cholesterol during early stages of infection and increases PV cholesterol later during PV maintenance. Given the role cholesterol plays in PV fusion with endosomes, as well as PV pH, *C. burnetii* most likely tightly regulates PV membrane cholesterol during the infectious cycle.

#### A. phagocytophilum

Infection of human premyelocytic leukemia cell line (HL-60) with *A. phagocytophilum* resulted in a 2-fold increase in total host cell cholesterol level (Xiong et al., 2009). Inhibitor studies and gene expression analyses revealed that *A. phagocytophilum* does not require *de novo* cholesterol synthesis but acquires cholesterol by upregulating LDLR at both the mRNA and the protein level (Xiong et al., 2009). Interestingly, *A. phagocytophilum* does not target SREBP, the primary transcription factor regulating *ldlr* expression. Instead, the 3′ end of the *ldlr* mRNA transcript is stabilized through an unknown mechanism (Xiong et al., 2009). However, the extracellular signal-regulated kinase (ERK) pathway appears to be involved, as ERK was upregulated during *A. phagocytophilum* infection, and inhibiting the upstream kinase MEK lowered *ldlr* expression levels and reduced bacterial infection (Xiong et al., 2009). How the bacterium targets this process still remains unknown, though it is clear that LDL uptake is essential for *A. phagocytophilum* pathogenesis.

### Cholesterol efflux

Excess cholesterol can be exported out of the cell by the ATP-binding cassette transporters ABCA1 and ABCG1. ABCG1 is found primarily in endosomes, while ABCA1 cycles between endosomes and the plasma membrane (Neufeld et al., 2001; Tarling and Edwards, 2011). While both ABCA1 and ABCG1 transfer cholesterol to a number of extracellular particles, ABCA1 promotes HDL assembly at the plasma membrane through binding ApoA-1, a main component of HDL (Phillips, 2013). By targeting host cholesterol efflux pathways, intracellular pathogens can further fine-tune host cholesterol to benefit bacterial growth.

#### *Chlamydia* spp.

*C. pneumoniae* infection decreased cholesterol efflux by downregulating expression of ABCA1 in multiple cell types including A549 lung epithelial cell lines (Korhonen et al., 2013), LDL-treated HUVEC cells (Sun et al., 2014), and THP-1 macrophage-derived foam cells (Zhao et al., 2014). ABCG1 was also downregulated in *C. pneumoniae*-infected HUVECs (Sun et al., 2014). Experimental measurement of cholesterol efflux to ApoA-1 showed a 50% decrease in *C. pneumoniae*-infected THP-1 macrophage-like foam cells compared to uninfected or heat-killed bacteria-infected cells (Zhao et al., 2014). Further, *C. pneumoniae* appeared to downregulate host cholesterol efflux by increasing microRNA miR-33 levels, which is produced from the SREBP intron and downregulates ABCA1 (Zhao et al., 2014). Upregulation of miR-33 was triggered by the innate immune pattern recognition receptor TLR2 (toll like receptor 2) which activates NF-kB-mediated upregulation of miR-33 upon bacterial recognition (Zhao et al., 2014). While viable organisms were required for this process, it is unknown if the bacteria are directly activating TLR2 or if this is strictly a host immune response. However, these data collectively indicate that *C. pneumoniae* targets efflux as a mechanism to further increase the levels of intracellular cholesterol.

#### C. burnetii

*C. burnetii* differentially regulated *apoE* and *apoC* gene expression in THP-1 macrophages (Ren et al., 2003; Mahapatra et al., 2010). In addition, a genome-wide RNA interference screen in HeLa cells revealed that siRNA depletion of apolipoproteins involved in lipid transport, including ApoA2, ApoC4, ApoL1, ApoL2, and ApoL5, affected the total number of *C. burnetii* PVs (McDonough et al., 2013). This suggests that cholesterol efflux may play an important role during *C. burnetii* infection, although the precise mechanisms and purpose are unknown.

### Cholesterol storage

Eukaryotic cells store excess cholesterol in lipid droplets (LD), specialized organelles comprised of a phospholipid monolayer surrounding a neutral lipid core of esterified cholesterol and triacylglycerols. Prior to packaging in ER-derived LDs, excess cholesterol is esterified by acyl CoA transferase (ACAT). LDs are coated by a special class of proteins called perilipins, which help prevent LD breakdown (Listenberger et al., 2007). LDs serve as an important source of lipids for membrane synthesis or energy metabolism, as well as immune modulators. Finally, LD accumulation leads to foam cell formation, a hallmark of atherosclerosis.

#### *Chlamydia* spp.

Atherosclerosis and foam cell formation play a significant role in *C. pneumoniae* pathogenesis. *C. pneumoniae* infection increased ACAT1 expression, and therefore esterified cholesterol, in THP-1 cells (Liu et al., 2010). Along with the decreased cholesterol efflux discussed earlier, this resulted in cholesterol accumulation within the host cell and promoted foam cell formation. The transcription factors LXR and the peroxisome proliferator-activated receptors PPARα and PPARγ, which regulate the expression of *acat1* and other cholesterol homeostasis genes such as *abca1* and *abcg1*, have been implicated in *C. pneumoniae*-induced foam cell formation (Chen et al., 2008; Naiki et al., 2008; Mei et al., 2009; Liu et al., 2010). For example, *C. pneumoniae* downregulated PPARα and PPARγ by targeting the c-Jun N-terminal kinase (JNK) branch of the MAP kinase pathway, leading to increased expression of *acat1, abca1* and *abcg1* and promoting foam cell formation (Mei et al., 2009; Liu et al., 2010). Treatment with PPARα and PPARγ agonists reversed this effect. Thus, *C. pneumoniae* induces foam cell formation by manipulating a signal transduction pathway that regulates both LD formation (ACAT1) as well as cholesterol efflux (ABCA1/G1).

During *C. trachomatis* infection, LDs were found inside the inclusion and the number of cytoplasmic LDs increased (Cocchiaro et al., 2008; Saka et al., 2015). LDs co-localized with the *C. trachomatis* inclusion protein IncA at the inclusion membrane and the lumen, suggesting that IncA participates in LD translocation into the inclusion (Cocchiaro et al., 2008). In addition, three *C. trachomatis* LD-associated proteins were identified: Lda1, Lda2, and Lda3 (Kumar et al., 2006). While the role of Lda1 and Lda2 have yet to be elucidated, Lda3 was localized to the inclusion, cytoplasmic LDs, and LDs within the inclusion (Cocchiaro et al., 2008). Lda3 overexpression decreased PLIN2 association with LDs, suggesting Lda3 replaces PLIN2 on LDs to promote LD translocation into the inclusion. While the role of LD translocation into the inclusion lumen is not clear, LDs could serve as a source of energy or membrane for intracellular *C. trachomatis* (Cocchiaro et al., 2008). As discussed earlier, the putative *C. trachomatis* cholesterol esterase (CT149) may hydrolyze cholesterol esters, freeing cholesterol for bacterial use (Peters et al., 2012). Further supporting the importance of LDs during *C. trachomatis* infection, inhibiting LD formation with either pharmaceutical inhibitors or gene knockouts significantly blocked *C. trachomatis* growth in epithelial cells and fibroblasts (Kumar et al., 2006; Peters and Byrne, 2015; Saka et al., 2015; Recuero-Checa et al., 2016). A recent proteomic analysis discovered that the LD proteome was altered during infection with *C. trachomatis*, with an enrichment of host lipid metabolism and biosynthesis proteins (Saka et al., 2015). Three additional bacterial inclusion proteins, Cap1, CTL0882, and IncG, were also found in the LDs. Together, these studies indicate that *C. trachomatis* actively manipulates LD formation, composition, and trafficking, potentially as a source of energy or lipids.

#### C. burnetii

*C. burnetii*-containing foam cells have been found in heart valves of an infected patient (Brouqui et al., 1994), and LDs were observed *in vitro* in the *C. burnetii* PV lumen of infected human alveolar macrophages (Graham et al., 2013). LD formation may increase during infection, as the expression levels of the LD coat protein PLIN2 and fatty acid binding protein FABP4, which transfers fatty acids to the ER for packaging in LDs, were upregulated in infected THP-1 cells (Ren et al., 2003; Mahapatra et al., 2010). Furthermore, siRNA depletion of patatin-like phospholipase domain-containing protein 2 (PNPLA2), the phospholipase involved in LD breakdown, led to an increased number of *C. burnetii* PVs in HeLa cells (McDonough et al., 2013). A similar observation was made following depletion of the long chain fatty acyl-CoA ligase ACSL6, which is important in neutral lipid synthesis (McDonough et al., 2013; Teodoro et al., 2016). In addition, treatment of monkey kidney epithelial cells (Vero cells) with an LD-localized broad spectrum antiviral molecule ST699 inhibited *C. burnetii* intracellular growth (Sandoz et al., 2014). These data point to a role for LD homeostasis during infection, though it has not been determined if LDs are indeed targeted by *C. burnetii* or if *C. burnetii* growth is altered when LD formation is blocked.

#### A. phagocytophilum

A gene expression profiling study in *A. phagocytophilum-*infected HL-60 cells revealed increased expression of the major LD protein PLIN1 (de la Fuente et al., 2005; Manzano-Roman et al., 2008). Further studies revealed PLIN1 expression increased with bacterial replication, and siRNA knockdown of PLIN1 led to a 50% decrease in *A. phagocytophilum* replication (Manzano-Roman et al., 2008). As PLIN proteins are critical for LD formation, LDs most likely play an important role during *A. phagocytophilim* infection (Tansey et al., 2004; Brasaemle, 2007).

#### *Rickettsia* spp.

*O. tsutsugamushi* induced LD formation in mouse L-929 fibroblast cells (Ogawa et al., 2014). Although the difference in cholesterol ester levels was not determined, lipid composition analysis revealed *O. tsutugamushi*-induced LDs were enriched in triacylglycerols and could serve as an energy source for the bacteria. While these studies suggest that *O. tsutsugamushi* induce LD accumulation, their contribution to bacterial intracellular growth is yet to be determined.

## Conclusion

**Figure 1:**
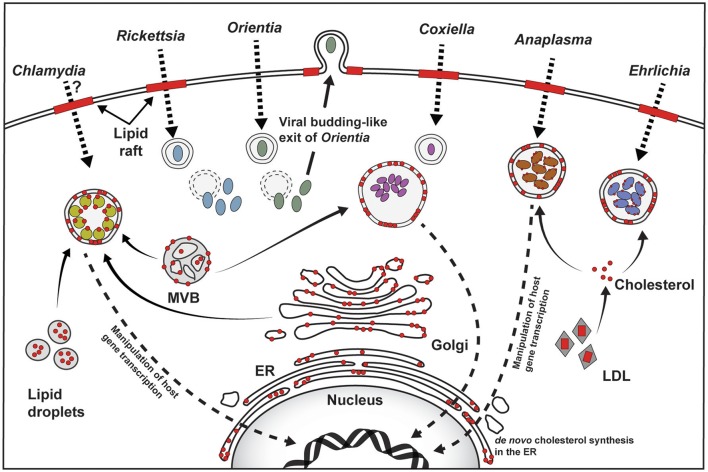
**Overview of host cholesterol-pathogen interactions**. For *Rickettsia, Coxiella, Anaplasma, and Ehrlichia*, attachment to the host cell plasma membrane and subsequent host cell entry involves cholesterol-rich lipid rafts (red bars). The role of lipid rafts for *Chlamydia* is unclear, and may be species- or host cell-specific, while *Orientia* utilizes lipid rafts for exit. Cholesterol (shown as red circles) is found in the pathogen-containing vacuoles, as well as the bacterial membrane of *Chlamydia, Anaplasma, and Ehrlichia*. Golgi-derived vesicles, multivesicular bodies (MVB), and lipid droplets traffic to the *Chlamydia* inclusion and serve as source of cholesterol. The source of cholesterol in the *Coxiella* vacuole is not clear, but may involve MVBs, while *Anaplasma* and *Ehrlichia* intercept LDL-derived cholesterol. *Chlamydia, Coxiella*, and *Anaplasma* target host gene expression to manipulate cholesterol homeostasis (Dotted arrow pointing to the nucleus).

For obligate intracellular bacteria, entry into the host cell and subsequent formation and maintenance of a vacuolar or cytoplasmic niche is essential for pathogen growth and survival. Due to cholesterol's multiple cellular functions, intracellular pathogens target cholesterol to obtain nutrients, membrane, or manipulate cellular signaling. This bacteria-host cholesterol interaction occurs at various stages of infection including host cell binding and internalization, niche formation, intracellular replication, and dissemination. The pathogens discussed in this review have appreciably different lifestyles with unique ways to manipulate host cell cholesterol (Figure 1).

*Chlamydia* spp. initially interact with cholesterol-rich lipid rafts during host cell entry, followed by targeting multiple host cholesterol trafficking pathways in order to establish the intracellular niche. Cholesterol is required in both the *Chlamydia* envelope and the inclusion membrane. While it has not been definitively demonstrated, cholesterol most likely plays a structural role in the bacterial envelope. Inclusion membrane cholesterol serves at least two purposes. First, inclusion cholesterol-rich microdomains contain both bacterial and host proteins involved in microtubule-dependent trafficking of the inclusion. Second, the cholesterol-binding protein caveolin may facilitate interactions between the inclusion and nutrient-rich endosomes and vesicles. In order to increase cholesterol availability, *Chlamydia* manipulates host cell cholesterol homeostasis. Both *C. trachomatis* and *C. pneumoniae* induce LD accumulation, which potentially provides lipids for bacterial growth. LDs appear particularly important to *C. trachomatis*, with bacterial proteins possibly facilitating translocation and breakdown of LDs in the inclusion lumen. *C. pneumoniae* reprograms cholesterol metabolism, increasing cholesterol levels that contribute to *C. pneumoniae*-mediated atherosclerosis and foam cell formation.

While *Chlamydia* spp. inclusions divert from the endocytic pathway, the *C. burnetii* niche is phagolysosome-like and highly fusogenic with host endosomes and autophagosomes. *C. burnetii* requires lipid rafts for entry into non-phagocytic cells, although the role of lipid rafts in macrophages has not been determined. Once inside the cell, the cholesterol requirement of *C. burnetii* appears complex and time-dependent. Due to the fusogenicity of the PV, it is likely that multiple sources of cholesterol traffic to the PV, though it is unknown how cholesterol trafficking or cholesterol levels change during PV development and maintenance. The bacteria are most sensitive to cholesterol during the initial stages of infection, before the PV is fully established. One possibility is that cholesterol regulates the *C. burnetii* T4SS, which secretes bacterial effector proteins necessary for PV expansion. Cholesterol also clearly influences PV pH, which is known to play an important role in *C. burnetii* metabolism. *C. burnetii* appears to have multiple mechanisms to manipulate host cholesterol levels, including targeting cholesterol efflux and storage. Further, *C. burnetii* may enzymatically modify cholesterol, or use membrane contact sites to transfer cholesterol from the PV to the host ER. Finally, our current knowledge of *C. burnetii* manipulation of host cholesterol is based on experiments with an avirulent strain, which contains truncated LPS compared to virulent bacteria. Studies with virulent bacteria will enable us to link host cholesterol to disease outcome during *C. burnetii* infection.

The importance of cholesterol during *A. phagocytophilum, E. chaffeensis*, and *Rickettsia* spp. infection is comparatively understudied. *A. phagocytophilum* and *E. chaffeensis* utilize lipid rafts during host cell entry. While *A. phagocytophilum* obtains cholesterol by hijacking the host LDL uptake and NPC1 trafficking, not much is known about *E. chaffeensis* and its effect on host cholesterol metabolism. Unlike the other discussed pathogens, *Rickettsia* spp. replicate freely in the cytoplasm and therefore have different cholesterol requirements than vacuolar pathogens. *R. conorii* targets cholesterol during entry, whereas *O. tsutsugamushi* requires it during egress from the host cell. Although *O. tsutsugamushi* infection increases LD accumulation, if and how these *Rickettsia* spp. target host cholesterol metabolism remains unknown.

Regardless of different bacterial life cycles and host pathways targeted, host cholesterol manipulation at varying stages of these intracellular life cycles seems to be the unifying theme (Table 1). Pathogen-mediated manipulation of host cell cholesterol metabolism still remains understudied, with a focus on gene expression analysis and little functional data. An additional limitation is a lack of *in vivo* data, which will be critical to fully understanding the role of cholesterol during infection. Finally, several questions still remain unanswered: (1) Is cholesterol manipulation cell type-dependent? (2) Can other sterols substitute for cholesterol? (3) What is the advantage of incorporating host cholesterol in the bacterial membrane? (4) What are the bacterial proteins responsible for manipulating host cholesterol? (5) Is disease outcome influenced by the patient cholesterol levels? Answering these and other questions will provide significant insight into the role of cholesterol during pathogenesis of obligate intracellular bacteria.

**Table 1:** **Summary of manipulation of host cholesterol by obligate intracellular bacterial pathogens**.

**Bacteria**	**Utilize lipid raft/caveolae for entry or exit**	**Cholesterol-rich microdomains on vacuole membrane**	**Cholesterol trafficking to vacuole**	**Bacterial manipulation of host cholesterol homeostasis**		
				**Uptake**	**Efflux**	**Storage**
*Chlamydia* spp.	Controversial	Yes	Yes	Yes	Yes	Yes
*Coxiella burnetii*	Yes (entry)	Yes	Yes	Yes	Yes	Yes
*Anaplasma phagocytophilum*	Yes (entry)	Unknown	Yes	Yes	Unknown	Yes
*Ehrlichia chaffeensis*	Yes (entry)	Unknown	Yes	Unknown	Unknown	Unknown
*Rickettsia conorii*	Yes (entry)	N/A	N/A	Unknown	Unknown	Unknown
*Orientia tsutsugamushi*	Yes (exit)	N/A	N/A	Unknown	Unknown	Yes

## Author contributions

All authors listed have made substantial, direct and intellectual contribution to the work, and approved it for publication.

### Conflict of interest statement

The authors declare that the research was conducted in the absence of any commercial or financial relationships that could be construed as a potential conflict of interest.
